# Exercise as A Potential Therapeutic Target for Diabetic Cardiomyopathy: Insight into the Underlying Mechanisms

**DOI:** 10.3390/ijms20246284

**Published:** 2019-12-12

**Authors:** Dae Yun Seo, Jeong Rim Ko, Jung Eun Jang, Tae Nyun Kim, Jae Boum Youm, Hyo-Bum Kwak, Jun Hyun Bae, Amy Hyein Kim, Kyung Soo Ko, Byoung Doo Rhee, Jin Han

**Affiliations:** 1 National Research Laboratory for Mitochondrial Signaling, Department of Physiology, BK21 Plus Project Team, College of Medicine, Cardiovascular and Metabolic Disease Center, Inje University, Busan 47392, Korea; sdy925@gmail.com (D.Y.S.); kjrsos0217@gmail.com (J.R.K.); venus724@hanmail.net (J.E.J.); kimtn031@gmail.com (T.N.K.); youmjb@inje.ac.kr (J.B.Y.); amyhikim@gmail.com (A.H.K.); kskomd@paik.ac.kr (K.S.K.); bdrhee@hanmail.net (B.D.R.); 2 Department of Kinesiology, Inha University, Incheon 22212, Korea; kwakhb@inha.ac.kr; 3 Institute of Sport Science, Seoul National University, Seoul 08826, Korea; baexx068@snu.ac.kr

**Keywords:** diabetes, exercise, cardiomyopathy, heart failure

## Abstract

Diabetes mellitus is associated with cardiovascular, ophthalmic, and renal comorbidities. Among these, diabetic cardiomyopathy (DCM) causes the most severe symptoms and is considered to be a major health problem worldwide. Exercise is widely known as an effective strategy for the prevention and treatment of many chronic diseases. Importantly, the onset of complications arising due to diabetes can be delayed or even prevented by exercise. Regular exercise is reported to have positive effects on diabetes mellitus and the development of DCM. The protective effects of exercise include prevention of cardiac apoptosis, fibrosis, oxidative stress, and microvascular diseases, as well as improvement in cardiac mitochondrial function and calcium regulation. This review summarizes the recent scientific findings to describe the potential mechanisms by which exercise may prevent DCM and heart failure.

## 1. Introduction

The prevalence of type 2 diabetes (T2DM) is a primary concern worldwide, and its incidence is increasing at an alarming rate [1]. T2DM leads to the impairment of both health and quality of life, causing chronic heart disease, cardiovascular disease, microvascular complications, and neurological abnormalities [2,3]. Addtionally, T2DM is associated with severe arteriosclerosis, hypertension, hyperlipidemia, and inflammation, all of which further contribute to the development of cardiovascular disease [4]. Cardiovascular disease, which is the primary complication in patients with diabetes mellitus, progresses to heart failure in more than 70% of cases, thereby drastically increasing the mortality and morbidity rate [5].

Recent molecular studies have suggested that the development of diabetic cardiomyopathy (DCM) involves mitochondrial dysfunction, fibrosis, oxidative stress, Ca^2+^ dysregulation, and microvascular dysfunction [6,7,8]. Numerous studies have shown that exercise can prevent cardiac injury and improve mitochondrial biogenesis in DCM [9,10,11]. Despite its broad clinical and pre-clinical impact, the mechanism underlying the protective effects of exercise on T2DM and DCM remains poorly understood. 

Regular exercise has undisputed benefits for diabetes. It helps to improve glucose and insulin metabolism and effectively decreases the risk factors for cardiovascular diseases. Additionally, increased physical activity and regular exercise helps to improve cardiopulmonary fitness and reduce mortality and the development of DCM in type 2 diabetes [12]. Scientific literature suggests that the frequency and intensity of exercise are important to mitigate the causes of DCM. This review summarizes the mechanisms underlying the pathogenesis of DCM and the role of exercise as a potential therapeutic approach for the clinical management of DCM. 

## 2. Clinical Aspects of DCM

### 2.1. Definition and Characteristics of DCM

**Figure 1:**
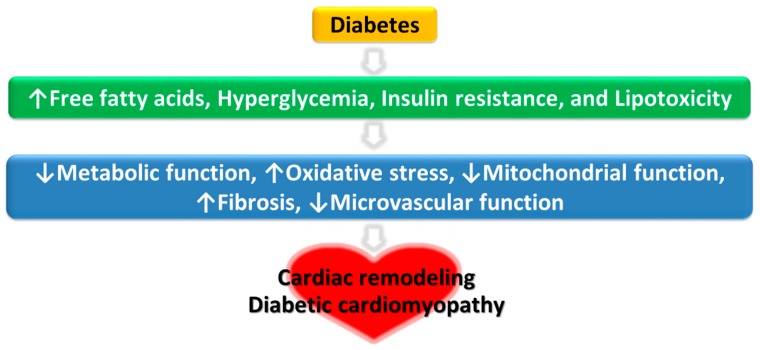
Multiple pathophysiological mechanisms of diabetic cardiomyopathy.

Diabetes-induced DCM is defined as a structural or functional myocardial dysfunction in the absence of coronary artery disease, hypertension, or valvular heart disease [13]. DCM is characterized by cardiac fibrosis, dysregulation of cardiac metabolism, and structural and functional changes of the left ventricle. These changes include left ventricular diastolic dysfunction, reduced left ventricular ejection fraction, pathological left ventricle hypertrophy, and interstitial fibrosis [14,15,16]. Collectively, these malfunctions increase the susceptibility of the heart to ischemic injury and the overall likelihood of developing heart failure. Left ventricular dysfunction with ventricular wall stiffness remains common in patients with well-controlled type 2 diabetes [17]. Additionally, DCM progression leads to reduced myocardial contractility and relaxation, ultimately leading to heart failure with reduced ejection fraction (HFrEF) [18]. The mechanisms underlying the development of DCM are summarized Figure 1 (adapted from [19]).

### 2.2. Treatment Strategies for DCM

Myocardial functional abnormalities associated with diabetes reportedly depend on hyperglycemia and dysregulated energy metabolism. Despite the well-known effects of drugs and other interventions on controlling diabetes and its associated complications, the strategy for comprehensive clinical management of DCM remains mostly unexplored. Therefore, therapeutic interventions for treating DCM have focused on controlling glucose and lipid levels, lowering blood pressure, and reducing coronary artery disease risk factors [20,21,22]. In recent years, pharmacological treatments of diabetes with beta-blockers (β-blockers), calcium (Ca^2+^) channel antagonists, and renin-angiotensin-aldosterone system inhibitors have been shown to impede coronary artery disease in T2DM [23,24]. Furthermore, metformin and sodium-dependent glucose transporter 2 (SGLT2) inhibitors are shown to be effective in treating DCM in animal models [25,26]. The results of recently completed SGLT2 inhibitor cardiovascular outcomes trials (CVOTs) have led to a paradigm shift in T2DM treatment from a glucocentric approach to the reduction of cardiovascular risk.

Interestingly, the reduction in cardiovascular risk in CVOTs SGLT2 inhibitors was observed much earlier than would be expected if the main mechanism of cardiovascular benefit was the result of an anti-atherosclerotic effect [27,28]. Suggested mechanisms are likely to be multifactorial and may include hemodynamic effects, such as reductions in BP and intravascular volume, as well as metabolic effects, such as changes in adiposity and fuel energetics. Other factors, such as possible effects on inflammation and nitric oxide, as well as potential CV and metabolic effects of increased glucagon release, may warrant further investigation [29]. These mechanisms might provide cardiovascular benefits through a range of effects on the heart, including reduction in left ventricular (LV) load and wall changes, attenuation of cardiac fibrosis and inflammation, and improvement in myocardial energy production [30].

Non-pharmacological interventions, such as increasing physical activity and regular exercise improve the health and quality of life in patients with obesity or T2DM and its associated complications [31,32]. Regular exercise is known to improve cardiac function and exert protective effects against cardiovascular disease and ischemic damage [33]. It also induces physiological cardiac hypertrophy through cardiac cell growth, and improves cardiac metabolism and function [34]. Furthermore, regular exercise inhibits cardiomyocyte apoptosis and myocardial fibrosis; it also improves hemodynamic impairment caused by T2DM-associated hyperglycemia [35]. Another study also reported that exercise improves systemic glucose regulation and insulin sensitivity while decreasing cardiac fibrosis and oxidative stress, thereby reducing the risk factors for cardiovascular disease [36]. Collectively, these studies indicate that exercise plays an essential role in the regulation of cardiac function and may be a potential target for the therapeutic management of DCM.

### 2.3. Clinical Significance of Exercise for the Treatment of DCM

The majority of published studies have reported that moderate to high-intensity exercise can increase the survival rate in patients with T2DM [37,38,39]. Hu et al. [40] reported that moderate to high-intensity exercise reduces the risk factors for cardiovascular diseases by lowering the body mass index (BMI) and blood pressure. Additionally, exercise duration was reported as an important factor for achieving the health benefits that minimize the risk factors for cardiovascular diseases. Karjalainen et al. [41] found that habitual and leisure-time physical activities decreased the risk factors for cardiovascular disease. However, home-based exercises did not significantly ameliorate cardiovascular disease risk factors [42,43]. According to the 2019 American Diabetes Association (ADA) guidelines, 150 min or more of moderate-to-vigorous intensity aerobic activity per week is recommended to harness the protective effects of exercise against diabetic complications [44]. Over the long-term, regular exercise of moderate-to-vigorous intensity is an effective strategy to prevent the development of DCM. However, it is difficult for diabetic patients to exercise on a long-term basis. Table 1 presents a summary of clinical studies on the preventive effects of exercise on diabetes-related cardiovascular diseases.

## 3. Potential Mechanisms of Protective Effects of Exercise Against DCM

### 3.1. Cardiac Cell Metabolism 

Impaired energy metabolism in the myocardium or “metabolic remodeling of the heart” can lead to structural changes in cardiomyocytes, eventually leading to cardiomyopathy [14]. Under normal physiological conditions, glucose transporter-4 (GLUT-4), an intracellular protein, is translocated to the cell membrane in response to insulin, where it facilitates glucose uptake and utilization [48,49]. However, in diabetes, the expression and function of GLUT-4 are compromised, leading to a marked reduction in glucose transport and impaired energy utilization in the myocardium [50]. However, many studies have reported that moderate exercise can reverse these effects by increasing GLUT-4 expression and glucose transport, thereby activating the pyruvate dehydrogenase complex [51]. Related findings suggest that exercise protects pancreatic beta cells by inducing the expression of insulin-sensitive adenosine monophosphate-activated protein kinase (AMPK) and preventing energy metabolism dysfunction in insulin-deficient diabetes mellitus [52]. Furthermore, exercise has been shown to reduce Forkhead box protein O1 (FOXO1) and FOXO1-related factors in pancreatic beta cells and in the myocardium of obese rats; it also activates insulin signaling pathways [53]. Additionally, exercise enhances the function of insulin-regulated glucose transporters via upregulating the expression of protein kinase C [54]. Therefore, exercise exerts its protective effects by inducing the pancreatic beta cells to increase insulin secretion, thereby activating the insulin signaling pathway, upregulating GLUT-4 expression, and enhancing intracellular energy metabolism.

Additionally, previous study has reported increased free fatty acid levels in patients with insulin resistance and T2DM [55]. Hence, this finding indicates that decreased glucose oxidation rates result in the use of fatty acid (FA) oxidation products as energy sources in the diabetic heart [55,56,57]. Consequently, the abnormal energy metabolism, which reduced glucose utilization and increased FA oxidation, was related to a primary deficit in glucose uptake, glycolysis inhibition, and decreased glucose oxidation [58,59]. However, exercise can improve glucose oxidation and stimulate a concomitant reduction in FA oxidation, thus promoting glucose use by the myocardium and cardiac function [11].

### 3.2. Calcium Regulation in Cardiac Cells

Calcium plays a pivotal role in muscle fiber differentiation, skeletal muscle contraction, cell signal transduction, and energy production [7]. Dysregulated Ca^2+^ homeostasis leading to myocardium contractile dysfunction is an important marker of DCM [60,61]. The reduced activity of the sarcoplasmic reticulum Ca^2+^-ATPase (SERCA2a) decreases the rate of Ca^2+^ transportation in the sarcoplasmic reticulum [60]. Patients with T2DM exhibit reduced Na^+^-Ca^2+^ exchange in the cardiomyocytes, whereas the SERCA2a retains its normal function [60,62,63]. This leads to the accumulation of Ca^2+^ ions in the sarcoplasmic reticulum. However, exercise improves the expression and activity of SERCA2a, and regulates Ca^2+^ levels via Ca^2+^ calmodulin-dependent protein kinase phosphorylation, thereby improving the myocardial contractile function [64]. Cassidy et al. [47] noted that intermittent, high-intensity exercise-mediated increases in myocardial contractility could be mimicked by restoring L-type Ca^2+^ channels, thereby increasing T-transverse tubule density, and enhancing Ca^2+^ release and excitation-contraction coupling.

### 3.3. Mitochondrial Function of Cardiac Cells

Mitochondria are important for energy metabolism, and the cardiac mitochondria processes the highest amounts of oxygen to meet the energy required for proper functioning of the organ. Therefore, mitochondrial dysfunction can play an important role in the pathogenesis of DCM [49]. The ultrastructural changes of the cardiac mitochondria associated with DCM include reduction of mitochondrial density, mitochondrial swelling, damage to the inner membrane, and increased mitochondrial matrix [17]. Moderate-intensity exercise has been shown to reduce these ultrastructural changes in diabetes and exerts a protective effect on the mitochondrial function [65]. Exercise regulates mitochondrial metabolism and activates downstream transcription factors such as PPAR gamma co-activator-1 alpha (PGC-1α) [66,67]; it also induces mitochondrial DNA replication, transcription, and mitochondrial biogenesis [68]. Furthermore, exercise improves the contractility of cardiomyocytes by regulating calcium cycling and ameliorates mitochondrial Ca^2+^ uptake [69]. In particular, resistance exercise improves mitochondrial efficiency and heart function via increase in PGC-1α and mitochondrial transcription factor A (TFAM) expression [10]. Furthermore, unlike moderate-intensity exercise, high-intensity exercise is reported to increase cardiac mitochondrial content. However, Veeranki et al. [65] found that moderate-intensity exercise prevents DCM-associated contractile dysfunction and restores mitochondrial function and connexin 43 levels in diabetic (db/db) mice models. These results suggest that exercise plays a vital role in cardiac mitochondrial metabolism and maximizes calcium efficiency. Hence, the enhancement of mitochondrial biogenesis in the diabetic myocardium may correlate with exercise intensity and warrants further research in this field.

### 3.4. Oxidative Stress in Cardiomyocytes

T2DM-associated hyperglycemia, chronic inflammation, and oxidative stress are linked with mitochondrial dysfunction and the development of DCM [70]. Hyperglycemia induces oxidative stress by enhancing the production of oxygen free radicals and facilitating cardiomyocyte apoptosis [24,71]. The excess oxygen free radicals interact with lipids, proteins, and DNA and lead to pathological changes [24]. However, studies have shown that exercise reduces the production of reactive oxygen species and alleviates oxidative stress-mediated damage [72]. Besides, exercise improves glucose metabolism in the diabetic myocardium and pancreas. Long-term regular exercise has been shown to decrease nicotinamide adenine dinucleotide phosphate (NADPH) oxidase, increase nitric oxide synthase and nitric oxide production, and enhance anti-oxidative activity in the endothelial cells [73]. The nuclear factor erythroid 2-related factor 2 (Nrf2) is crucial for defending cardiomyocytes against intracellular oxidative stress, and as a regulator of antioxidant response elements [74,75,76,77,78]. Cardiomyocytes of Nrf2 knockout mice are reported to suffer from oxidative stress. Exercise upregulates the expression of Nrf2, thereby mitigating oxidative stress [76]. However, it has been reported that low-intensity exercise reduces malondialdehyde, an indicator of oxidative stress, and upregulates the expression and function of anti-oxidant enzymes such as superoxide dismutase, glutathione peroxidase, and catalase in the myocardium [79]. These studies demonstrate that exercise exerts anti-oxidative effects by regulating the activity of enzymes, oxygen-free radicals, and nuclear factors; however, these effects depend on the intensity of exercise.

### 3.5. Myocardial Fibrosis

T2DM-induced accumulation of extracellular matrix (ECM) proteins in the heart, also known as myocardial fibrosis, is the most marked histopathological change associated with DCM [8,14,15,80,81,82,83,84]. Myocardial fibrosis leads to structural and functional changes in the heart through collagen deposition, interstitial fibrosis, and perivascular fibrosis [14]. However, several studies have reported that exercise can reduce myocardial fibrosis and promote cardiac structure remodeling, eventually improving cardiac function in DCM [10]. Exercise is also reported to reduce myocardial fibrosis by lowering blood pressure [11]. Moreover, exercise has been shown to increase matrix metalloproteinase-2 and collagen degradation, while inhibiting myocardial fibrosis in obese mice [80,85]. The interaction between collagen and glucose leads to the formation of advanced glycation end products, which induce endothelial dysfunction and arterial and cardiac cirrhosis [15]. Novoa et al. [64] reported that long-term, regular, high-intensity exercise exerts positive effects on cardiac structure remodeling by decreasing cardiomyocyte hypertrophy, collagen deposition, and myocardial fibrosis. Therefore, exercise-induced abrogation of myocardial fibrosis may be highly correlated with energy metabolism, including decreased blood glucose and glycogen deposition in the heart.

### 3.6. Apoptosis of Cardiomyocytes

T2DM-induced apoptosis of cardiomyocytes is one of the hallmark features of DCM. Hyperglycemia induces the activation of mitochondrial cytochrome c and promotes its release into the cytoplasm; it also activates caspase-3 and the downstream apoptotic pathway in cardiomyocytes [8]. These changes are key factors leading to cardiac hypertrophy, cardiac remodeling, and heart failure in DCM [17,86]. The c-Jun N-terminal kinases (JNKs) group of mitogen-activated protein kinases (MAPKs) are activated under the conditions of metabolic disorders and T2DM [20,87,88,89,90]. The JNKs promote apoptosis in cardiomyocytes by activating the pro-apoptotic proteins caspase 8 and Bax [72]. Veeranki et al. [65] showed that exercise improves the mitochondrial transmembrane potential and inhibits cytochrome c leakage into the cytoplasm, thereby preventing apoptosis of cardiomyocytes. Another study reported that exercise could reduce the phosphorylation of JNKs and inhibit the downstream apoptotic pathway in obese mice [91]. Additionally, exercise has been shown to increase the expression of B cell leukemia/lymphoma 2 (BCL-2) in cardiomyocytes of diabetic animal models. This result suggests that exercise can inhibit cardiac apoptosis or prevent the development of cardiac abnormalities [10]. Kanter et al. [92] reported the therapeutic effects of low-intensity exercise against apoptotic, biochemical, and morphological changes in cardiac tissues. Khakdan et al. [93] showed that high-intensity interval training increased the expression of Sirtuin 1 (Sirt1) and BCL-2 and significantly improved the left ventricular ejection fraction (LVEF) and fractional shortening (FS). Furthermore, studies have revealed that exercise improves diabetes via inhibition of endoplasmic reticulum (ER) stress-induced apoptosis; however, the extent of such inhibition depends on the intensity of exercise [9].

### 3.7. Microvascular Function of Cardiomyocytes

Hyperglycemia-induced microvascular changes are manifested as vascular endothelial defects and endothelial cell dysfunction, and they elicit a partial inflammatory response in the vascular endothelium [94]. These changes can affect glucose and insulin transport into cells, thereby inducing abnormal tissue function [18]. Decreasing levels of oxytocin (OT), arterial natriuretic peptides (ANP), and brain natriuretic peptides (BNP) in the heart are associated with DCM, indicating a role of cardiovascular hormones in db/db mice [95,96].

Exercise is shown to exert a protective effect on blood vessels, endothelial dysfunction [45], and the expression of OT, and natriuretic peptides in the diabetic heart [97], as it enhances the ventricular diastolic function [98]. It also improves the microvascular responses to insulin and insulin signaling. Exercise helps to maintain optimal blood vessel function while also sustaining the balance between hypotensive nitric oxide (NO) and hypertensive endothelin-1 via the insulin receptor substrate-1/phosphatidylinositol-3-kinase/protein kinase B (IRS-1/PI3K/AKT) and MAPK signaling pathways [89]. In both db/db mice and in diabetic patients, exercise training has been shown to have beneficial effects by decreasing corticosterone and catecholamine secretion. With exercise training, cardiac metabolism such as myocardial glucose uptake [31], glucose oxidation rates [99], and glycolysis [33] was improved in the db/db mouse model of T2DM. Additionally in db/db mice, exercise training increased glycogen synthase phosphorylation and decreased phosphorylation of 5’-AMP-activated kinase and downstream substrate acetyl-CoA carboxylase [100]. Although the glucose metabolic control in db/db mice remains yet to be clarified, exercise training had beneficial effects on nutrient metabolism [101] and myocardial substrate imbalance [31]. It supported the evidence that increased myocardial O-GlcNAcylation had a key role in the development of insulin resistance [102] and in protecting DM [103].

Moreover, Moien-Afshari et al. [104] reported that exercise training for 7 weeks upregulates and improves NO generation via production of endothelial nitric oxidative synthase protein and superoxide dismutase (SOD), which can consequently reverse endothelial dysfunction in the db/db mouse model of T2DM. Laher et al. [105,106] reported that 6 weeks of running wheel exercise had beneficial effects on endothelial function without reduction in plasma C-reactive protein levels, or any effect on blood glucose or insulin levels in diabetic mice. They also suggested that 8 weeks of aerobic exercise improves diabetic cardiac function through changes in titin-dependent myocardial stiffness [107]. Broderick et al. [97] suggested that 8 weeks of exercise training improved expression levels of cardioprotective genes such as *OP, ANP, BNP,* and *GATA4*, which regulate the synthesis of OT, ANP and BNP in the heart. Also, chronic oxytocin treatment for 12 weeks in db/db mice partially improved glucose and fat metabolism, and consequently ameliorated abnormal cardiac structural remodeling, thus, preventing cardiac dysfunction [108]. Interestingly, Gutkowska et al. [96] also stated that the effect of exercise seemed to have no direct correlation to the improvement of the hyperglycemic state of db/db mice, as they observed that the hyperglycemic state and body weights were not improved in the db/db exercise training group when compared to the control, suggesting a potential impairment of the cardioprotective system in diabetes that may stand independently of obesity and hyperglycemia. Nevertheless, exercise training had positive effects on the overall myocardial alternations in the hearts of patients with T2DM. While the nature of such mechanistic changes that occur with exercise and their correlation to DCM complications, especially that of the metabolic glucose control and correlation to hyperglycemia in diabetes, are yet to be clarified and still warrant further investigation, exercise training overall proved to have positive effects on both the molecular and vascular levels in improving cardiac function. Thus, exercise poses a prospective noninvasive and preventative therapeutic approach for diabetic cardiomyopathy. Details of the exercise-mediated pre-clinical studies and the potential mechanisms of the protective role of exercise on DCM are presented in Figure 1 and Table 2.

## 4. Prospective New Biomarkers in DCM

The pathophysiologic mechanisms of DCM are multifactorial processes that include altered cardiac cell metabolism, impaired calcium regulation, mitochondrial dysfunction, increased oxidative stress, altered myocardial fibrosis, higher induction of apoptosis, and microvascular disease. Exercise can protect the myocardium by altering these mechanisms for the better. However, there are no known biomarkers to distinguish patients with DCM, although there are several biomarkers such as natriuretic peptides that may be helpful in the diagnosis of heart failure. Cardiac injury markers including C-reactive protein and troponins are released in relation to different diseases such as myocardial infarction, myocarditis, or any secondary cardiac injury. These markers have not provided valuable information for early detection of DCM in the clinical setting. Thus, new biomarkers must be identified for early detection of cardiac responses such as hypertrophy, contractibility, steatosis, or even fibrosis [111].

Cardiotrophin-1 (CT-1), a member of interleukin-6 family, was originally isolated for its ability to induce a hypertrophic response in neonatal cardiac myocytes [112]. CT-1 is mostly released from cardiomyocytes after oxidative and mechanical stress or renin-angiotensin-aldosterone system stimulation [113]. CT-1 is known to modulate cardiac hypertrophy, contractility, fibrosis and ischemia through reduction of cell proliferation, apoptosis, oxidative stress and inflammation, and by activation of JAK/STAT and MAPK pathways [113,114]. A very interesting field of study is the physiological and reversible cardiac hypertrophy adaptive response to exercise. In one study, CT-1 levels were examined in a small and select cohort of elite athletes. Although there were no differences between basal circulating CT-1 levels in well trained athletes and controls, CT-1 levels during exercise were significantly different in the trained athlete group compared to the control group [115].

With regard to prospective new biomarkers related to the contractile function of the heart in DCM, Activin A, a molecule secreted by epicardial adipose tissue, may induce contractile dysfunction and insulin resistance in cardiomyocytes [116]. Circulating Activin A levels were negatively associated with glucose metabolism in cardiomyocytes, and positively with left ventricular mass/volume-ratio, reflecting a potential harmful effect on early diabetic cardiomyopathy in patients with T2DM [117]. Long-term regular exercise is associated with reduced risk for the development of metabolic and cardiovascular diseases. Although plasma Activin A levels increased after a single exercise session (45 min) [118], there is very limited information about the circulating profile of this molecule with long-term exercise and its role in the mechanisms of DCM.

Normal fat accumulation in the myocardium may be a protective response to provide a store of fuel for subsequent oxidation, whereas in DCM, a chronic imbalance between lipid storage and lipid oxidation may lead to cardiac dysfunction [119]. Steatosis-related factors released from the heart may be useful for early DCM detection. Heart-type FA binding protein (H-FABP) is a cardiac cytosolic protein that transports fatty acids to the mitochondria. It is up-regulated and located at the sarcolemma after lipid delivery [120]. H-FABP might be released into the blood after myocardial injury even though it is undetectable in healthy subjects. The presence of H-FABP has been observed in patients with T2DM in the early stages of cardiac injury. [121]. Conversely, H-FABP has been used as an indicator of FA utilization in the heart. Compared to the control group, rabbits with myocardial infarction (MI) group had down-regulated cardiac expression of H-FABP. Hence, exercise training increased H-FABP expression in the MI group, whereas it had no effect on H-FABP expression in the control group [122]. These results indicate that MI decreased cardiac fatty acid utilization, and that exercise could improve it. Most previous articles have discussed the diagnostic or prognostic role of H-FABP in a specific clinical diagnosis such as acute coronary syndrome or acute pulmonary embolism [123]. However, future studies should address the potential role of circulating H-FABP levels as an indicator of early DCM and whether the H-FABP biomarker is suitable to evaluate the beneficial effects of exercise training in patients with DCM. 

Likewise, chronic hyperglycemia facilitates the reaction of glucose with collagen to form advanced glycation end-products that promote the crosslinking of collagen molecules to produce fibrosis [124]. Fibrotic components may be an important link to the pathologic mechanism of DCM. Insulin-like growth factor binding protein-7 (IGFBP-7), which modulates insulin receptor activity by interaction with insulin growth factor-1, has been evidenced as a serum biomarker for diastolic dysfunction associated with vascular remodeling and cardiac hypertrophy and fibrosis in metabolic syndrome [125]. In addition, IGFBP-7 is associated with the severity of diastolic dysfunction [126]; it is also involved in fibrogenesis in patients with diabetes [127]. Consequently, IGFBP-7 levels increase in diabetes patients with normal diastolic function, and in patients with diastolic dysfunction either with or without diabetes; the highest values of IGFBP-7 levels are found in patients with diastolic dysfunction and diabetes [128]. 

Recently, cardiac microRNAs have been discovered as key modulators in the pathogenesis of DCM [129]. Circulating microRNAs present in the blood are extremely stable and may be potentially used as diagnostic and prognostic biomarkers for diabetic macrovascular complications as well as DCM [130]. Indeed, a number of microRNAs in plasma or whole blood have been reported in diabetic individuals. The microRNA-targeted mRNAs are altered in DCM. These microRNAs exhibit essential roles in regulating genes related to the aforementioned pathophysiologic pathways of DCM including hypertrophy, apoptosis, and fibrosis. MicroRNA-9, 21, 29, 30d, 34a, 144, 150, 320, and 378 correlated strongly with DCM and could be used as potential serum biomarkers in patients with DCM [131]. Although it is not clear whether exercise can normalize certain microRNAs expression in the myocardium, it is exciting to consider that exercise might be able to restore the expression of some microRNAs through cross-talk effect. For example, T2DM mice during a 10-week swimming exercise regime exhibited increase in the expression of microRNA-133 in cardiac tissue, improved contractile function, and decreased extracellular matrix regulator protein metallopeptidase-9 [132]. Since microRNA-133 is reportedly expressed and enriched in both cardiac and skeletal muscles [133]. Therefore, it is possible that after exercise skeletal muscles may secrete specific microRNAs into the circulation, which then travel to the cardiomyocytes to suppress fibrotic markers and reduce cardiac hypertrophy. The noticeable alterations in the microRNA profiles detected in the myocardium and the circulation, mirrors the underlying molecular pathology of DCM, which suggests that microRNAs may be effective biomarkers.

## 5. Clinical Perspective on the Future Use of Exercise in DCM Prevention

### Exercise Is an Early Diagnostic Tool for Prevention and Better Treatment of DCM 

Although recent research has advanced our understanding of the protective effects of exercise on the pathophysiology of DCM, there are no specific guidelines regarding the inclusion of exercise as a preventive or therapeutic modality for DCM clinical management. Even though the diastolic cardiac dysfunction is considered as the key step in DCM development [134,135], patients in the early stages of DCM generally do not present any clinical symptoms or signs [136,137]. Therefore, the majority of DCM patients remain asymptomatic until the later stages which are accompanied by systolic failure, at which point treatment modalities, including exercise, are ineffective. Since physical activity is restricted in the later stages of DCM [10], early diagnosis and proper preventive measures, including exercise, are crucial for both prevention and better prognosis of DCM.

It has been proposed that exercise during chemotherapy mitigates the doxorubicin-induced cardiomyopathy by restoring mitochondrial quality and redox signaling pathways [91]. Results from animal models have proven the effectiveness of exercise in preventing acute toxicity from a single dose of doxorubicin treatment [72,109]. Similar studies have shown that long-term exercise could be an effective adjuvant treatment strategy against doxorubicin-related cardiac toxicity [92,110]. Recently, despite the limitations of a clinical human trial, the American Heart Association has suggested that personalized exercise therapy could be considered as a part of precision medicine for cancer patients to improve cardiovascular mortality [138,139]. Based on these recommendations, exercise may be presumed to influence an improved outcome in patients with DCM. If biomarkers or non-invasive imaging modalities could be used for early identification of high-risk patients [46,140], it would be feasible to prescribe personalized exercise programs based on risk stratification. Since exercise may not only mitigate cardiac dysfunction but also improve the outcome of T2DM [36,134,141,142,143], a therapeutic approach with a tailored exercise prescription would positively impact DCM management in the future. To this end, the development of tools for early diagnosis and optimization of risk stratification models would be pivotal and in conjunction with well-designed clinical trials could provide the evidence to facilitate timely implementation.

## 6. Conclusions

**Figure 2:**
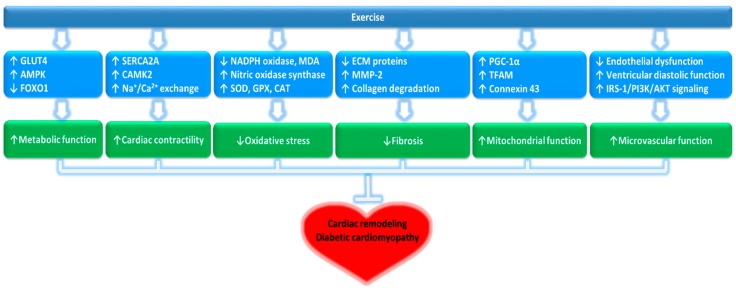
Potential molecular mechanisms of exercise influence on DCM.

Exercise is essential to maintain overall human health. Regular exercise is useful in the management of T2DM by mitigating the risk factors of associated comorbidities, including cardiovascular disease [36,43,143]. More importantly, exercise is a non-pharmacological intervention that can improve the outcome in patients with DCM by regulating cardiac mitochondrial metabolism, alleviating oxidative stress damage, and improving myocardial fibrosis, apoptosis, and vascular disorders [10,11]. Collectively, these possibilities suggest that exercise may have a potential role for inclusion in combination treatments for the prevention and management of DCM. The potential molecular mechanisms of how exercise can control DCM are summarized in Figure 2. However, future studies are warranted to provide molecular and clinical evidence, before exercise can be included in the clinical management of DCM.

## Figures and Tables

**Table 1:** A summary of clinical studies on the preventive effects of exercise in diabetes-related cardiovascular diseases.

Study Type (Design)	Subjects	Exercise Intervention				Effects	Ref
		Type	Frequency	Time (min)	Duration		
Human (non-RCT)	Patients with T2DM	Home-based exercise training (Rowing ergo meter)	3–4 days/wk	30 m every other day for a total of 28 sessions	8 wks	↑Insulin sensitivity ↑Forearm endothelial function ↓Plasma adhesion molecules (ICAM-1 & VCAM-1)	[43]
Human (RCT)	Patients with T2DM	Aerobic & Resistance Exercise	3–5 days/wk	75 min/session	12 wks	↑Anti-atherosclerotic effects ↓Cardiovascular events ↑Flow-mediated endothelium-dependent vasodilation	[45]
Human (non-RCT)	Patients with T2DM	Aerobic Exercise	4 days/wk	4–7 h/day	24 wks	↓Hepatic triglyceride ↓Paracardial fat volume	[46]
Human (RCT)	Patients with T2DM	High intensity interval training	NA	NA	12 wks	↑Left ventricular mass (g) ↑Early diastolic filling rate change (ml/s) ↑Peak torsion change ↓Body weight (kg) ↓Liver fat (%) ↓ALP (U/I) ↓HbA1c (%) ↓2–hr glucose (pmol/l) ↓2–hr AUGC (mmol/l)	[47]

↑, up; ↓, down; ALP, alkaline phosphatase; HbA1c, hemoglobin A1c; AUGC, area under the blood glucose curve.

**Table 2:** A summary of pre-clinical studies on the preventive effects of exercise in diabetes-related cardiovascular diseases.

Subjects	Exercise Intervention				Effects	Ref
	Type	Frequency	Time (min)	Duration		
STZ-diabetic rats (single injection of STZ: 40mg/kg)	Aerobic exercise	5 days/wk	60 min/day	12 wks	↑Ejection fractions (%) ↑Left ventricular end-systolic volume ↓Serum cTn-I levels ↓Apoptotic myocardial cells ↓GRP78, CHOP, cleaved caspase-12 protein	[9]
Obese diabetic mice (db/db)	Aerobic Exercise	5 days/wk	330m run at speed of 10 m/min (2 wks) + 330m run at speed of 11 m/min (3 wks)	5 wks	↑Body weight (gm) ↑Mean blood pressure (mmHg) ↑Heart weight (gm) ↑Blood glucose (mg/dL) ↑Stroke volume (µL) ↑Ejection fraction (%) ↑Fractional Shortening (%)	[65]
STZ-diabetic rats (single injection of STZ: 55 g/kg)	Aerobic Exercise	5 days/wk	10–15 min/day, total 60 min (speed 2m/min at grade 5%)	7 wks	↑Citrate synthase activity (µmol/tissue(g)/min) ↑Heart Rate (bpm) ↓Left ventricular end diastolic diameter & Left ventricular end systolic diameter (mm) ↑Fractional shortening (%) ↑Ejection fraction (%) ↑Cardiac output (ml/min) ↓QRS interval (m/sec) ↑Myocyte contractile kinetics velocity(µm/sec), extent of cell shortening (µm), and relengthening velocity (µm/sec) ↓Myocyte contractile kinetics time to 50% peak contractile velocity (m/sec) & time to 50% peak relaxation velocity (m/sec)	[63]
7 wk old diabetic rats (Injection STZ 65 mg)	Aerobic Exercise	5 days/wk	60 min/day (20 m/min pace)	9 wks	↑Cytoplasmic area (% of intracellular area) ↑Lipid droplets(µm^2^) ↑Mitochondria area, myofibrillar area, mitochondria quality index, cytoplasm area, and collagen fiber circumference	[69]
3 month old male Sprague Dwaley rat (Single dose of alloxan; 200 mg/kg)	High intensity of Aerobic Exercise	5 days/wk	NA	4 wks	↓Body weight (g) ↑Plasma glucose (mg/dL) ↑NOX2 & NOX4, p67^phox^ ↓SERCA2 ↓eNOS & BH_4_	[64]
BBDR (Biomedical Research Models) male rats	Aerobic Exercise	5 days/wk	50 min/day	8 wks	↑Plasma glucose (mg/dl) & HbA1c (%) ↑LV end-systolic volume (µl), LV end-diastolic volume (µl), and LV – dp/dt (mmHg/s) ↑Myocardial mitochondria fractional area (%)	[54]
Adult Sprague-dawley male rats + doxorubicin (DOX: 20 mg/kg body weight)	Aerobic Exercise	5 days/wk	60 min/day (30 m/min)	NA	↑Protect against Dox-mediated damage in mitochondria ↓Caspase 3 & calpain ↑SOD1, SOD2, Catalase, GPX1, catalase, and HSP72 ↓Hydrogen peroxide (pmol/mg/min)	[109]
8 wks old female C57BL6 mice	Aerobic Exercise + Resveratrol supplementation	5 days/wk	30 min/day	8 wks	↑Left ventricle posterior wall (mm) ↑Intraventricular septum (mm) ↑Left ventricle internal dimension (mm) ↑Fractional shortening ↓Citrate synthase activity (mmol/mg/min) ↓ANF & UCP3	[110]
Sprague-dawley (SD) diabetic rats	Aerobic Exercise	5 days/wk	60 min/day Low intensity: 20m/min High intensity: 34 m/min	12 wks	↓Left ventricular & diastolic volume ↓caspase 3, cTn-1 (lg/l), Grp78, CHOP, and Caspase 12	[9]
Sprague-Dawley rats (intravenous injection of streptozotocin: 60mg/kg	Aerobic Exercise	5 days/wk	60 min/day (32 m/min)	10 wks	↓Body weight (g) ↓Glucose (mmol/L) ↓Triglycerides (mmol/L) ↑Glucose oxidation (nmol/g/min) ↑Glycolysis (nmol/g/min) ↑Aortic flow (ml/min)	[33]
6-8 wk male Wistar rat + Doxorubicin (20 mg/kg)	Aerobic Exercise	5 days/wk	60 min/day	14 wks	↑Mitochondrial respiration, calcium tolerance, oxidative damage, and heat shock proteins ↓State 3 respiration, respiratory control ratio, uncoupled respiration, aconitase activity, and protein sulfhydryl content	[72]

↑, up; ↓, down; GRP78, glucose-regulated protein 78; CHOP, transcriptional induction of C/EBPhomologous protein; SSBP1; single-stranded DNA-binding protein1; PGC-1α, peroxisome proliferator-activated receptor gamma coactivator 1-alpha; NRF1, nuclear respiratory factor 1; TFAM, mitochondrial transcription factor A; TFB2M, Mitochondrial dimethyladenosine transferase 2; NOX2, NADPH oxidase 2; NOX4, NADPH oxidase 4; SERCA2, sarco/endoplasmic reticulum Ca2+-ATPase; eNOS, endothelial nitric oxide synthase; BH_4,_ tetrahydrobiopterin; HbA1c, hemoglobin A1c; LV, left ventricle; SOD1, superoxide dismutase 1; SOD2, superoxide dismutase 2; GPX1, glutathione peroxidase 1; HSP72, heat shock protein 72; ANF, atrial natriuretic peptide; UCP3, uncoupling protein.
